# Inonotus obliquus (chaga) ameliorates folic acid-induced renal fibrosis in mice: the crosstalk analysis among PT cells, macrophages and T cells based on single-cell sequencing

**DOI:** 10.3389/fphar.2025.1556739

**Published:** 2025-03-14

**Authors:** Yueling Peng, Yaling Zhang, Rui Wang, Xinyu Wang, Xingwei Liu, Hui Liao, Rongshan Li

**Affiliations:** ^1^ Department of Nephrology, Fifth Hospital of Shanxi Medical University (Shanxi Provincial People’s Hospital), Taiyuan, China; ^2^ Department of Nephrology, Taiyuan Central Hospital, Taiyuan, China; ^3^ Drug Clinical Trial Institution, Shanxi Provincial People’s Hospital (Fifth Hospital of Shanxi Medical University), Taiyuan, China

**Keywords:** renal fibrosis, single-cell RNA sequencing, traditional Chinese medicine, proximal tubular cells, macrophages, t cells

## Abstract

**Background:**

Renal fibrosis, characterized by the abnormal accumulation of extracellular matrix in renal tissue and progressive loss of kidney function, is posing a significant challenge in clinical treatment. While several therapeutic options exist, effective treatments remain limited. Inonotus obliquus (Chaga), a traditional medicinal mushroom, has shown promising effects in chronic kidney disease (CKD), yet its cellular and molecular mechanisms remain largely unexplored.

**Methods:**

We analysed the chemical composition of Chaga using UPLC-MS and predicted its biological targets using PubChem and Swiss Target Prediction. We used single-cell RNA sequencing to study cellular responses in a mouse model of folic acid-induced renal fibrosis, complemented by spatial transcriptomics to map cellular location patterns. Histological assessment was performed using H&E and Masson trichrome staining.

**Results:**

For the first time, we employed single-cell RNA sequencing technology to investigate Chaga treatment in renal fibrosis. Histological analysis revealed that Chaga treatment significantly reduced renal tubular damage scores [from 5.00 (5.00, 5.00) to 2.00 (2.00, 2.00), p < 0.05] and decreased collagen deposition area (from 11.40% ± 3.01% to 4.06% ± 0.45%, p < 0.05) at day 14. Through analysis of 82,496 kidney cells, we identified 30 distinct cell clusters classified into eight cell types. Key findings include the downregulation of pro-inflammatory M1 macrophages and upregulation of anti-inflammatory M2 macrophages, alongside decreased T cell responses. Single-cell sequencing revealed differential gene expression in proximal tubular subpopulations associated with reduced fibrosis. Pathway and network pharmacology analyses of 60 identified compounds in Chaga and their 675 predicted targets suggested potential effects on immune and fibrotic pathways, particularly affecting Tregs and NKT cells. Cell-to-cell communication analyses revealed potential interactions between proximal tubular cells, macrophages, and T Cells, providing insights into possible mechanisms by which Chaga may ameliorate renal fibrosis.

**Conclusion:**

Our study provided new insights into the potential therapeutic effects of Chaga in renal fibrosis through single-cell sequencing analysis. Our findings suggest that Chaga may represent a promising candidate for renal fibrosis treatment, though further experimental validation is needed to establish its clinical application.

## Introduction

Renal fibrosis is a grave renal pathology characterized by aberrant fibrogenesis within the kidney tissue, leading to a progressive loss of function (Huang et al., 2023). This condition represents the final common pathway of virtually all chronic kidney disease (CKD), affecting approximately 10% of the global population. The progression of renal fibrosis involves complex pathological processes, including activation of inflammatory cells, production of pro-fibrotic cytokines, and transformation of various kidney cells into myofibroblasts (Ruiz-Ortega et al., 2022; Mencke et al., 2017). It is therefore crucial to identify new therapeutic strategies and to understand the molecular basis of fibre formation.

Recent studies have highlighted the critical role of cellular crosstalk in the progression of renal fibrosis. Particularly, the interactions between proximal tubular cells, immune cells (including macrophages and T cells), and fibroblasts orchestrate the fibrotic process (Liang et al., 2024; Tang et al., 2019). Understanding these complex cellular interactions is crucial for developing targeted therapeutic strategies.

Chaga, a traditional medicinal mushroom, has garnered increasing attention for its therapeutic potential in various diseases. It is reported to exhibit immunomodulatory properties, and our primary studies in rodent models have shown its efficacy in improving the prognosis of diabetic nephropathy (Kou et al., 2021; Fordjour et al., 2023; Zhang et al., 2021). However, the cellular and molecular mechanisms underlying anti-fibrotic effects remain largely unexplored. To better understand the onset and progress of fibrosis and the efficacy of Chaga, we employ single-cell sequencing technology that offers unprecedented precision and scope in conventional medicine (Jovic et al., 2022; Papalexi and Satija, 2018). This technology allows for a detailed analysis of gene expression profiles across different cell subtypes within renal tissue, furnishing novel therapeutic perspectives from both gene target and immune cell subpopulation standpoints. Additionally, we have harnessed network pharmacology to discern how the molecular constituents of Chaga impact specific cell subtypes, providing insights into its mechanism of action.

Therefore, we aim to explore the transcriptomic alterations in proximal tubular epithelial cells, macrophages, and T cells during the process of renal fibrosis and Chaga treatment. By integrating single cell sequencing and network pharmacology approaches, we aspire to identify potential cellular responses and molecular changes that may contribute to understanding the effects of Chaga on renal fibrosis, potentially offering new directions for future therapeutic investigations.

## Materials and methods

### Sample preparation and UPLC-MS analysis

The Chaga specimen was procured from the Department of Pharmacy at the Fifth Hospital of Shanxi Medical University. Details of the extraction and preparation of Chaga are available in our previous publication (Zhang et al., 2021). Firstly, 50 mg of Chaga was dissolved in a methanol-water mixture (1:1, v/v) and used in preparation for ultra performance liquid chromatography-mass spectrometry (UPLC-MS) analysis. Secondly, this solution wa centrifuged at 17,000 g for 30 min at 20°C. The supernatant was concentrated at 35°C and subsequently re-suspended in 100 µL of 50% methanol. A further centrifugation step was conducted, thereby rendering the sample suitable for subsequent analysis. The mobile phase comprised 0.1% formic acid in water (solvent A) and acetonitrile (solvent B), implemented through a gradient elution protocol. The flow rate was consistently maintained at 0.4 mL/min, and the column temperature was set at 40°C. Mass spectrometric analysis was executed using an AB Sciex 5,600 Triple TOF system. This system was operated in a positive ion mode with an iron source voltage set to 5000 V, under the governance of Analyst TF 1.7 software. Data acquisition occurred in an Information Dependent Acquisition (IDA) mode, covering a mass-to-charge (m/z) range from 50 to 1,200. The data processing phase involved converting raw spectral data into. mzXML format using MassHunter and Proteowizard software. Subsequently, peak identification and alignment were conducted using MS-DIAL software. The acquired data were cross-referenced with established databases To ensure the accuracy of the analysis.

### Predicted biological targets of chaga compounds

We ascertained 60 chemical constituents of Chaga utilizing UPLC-MS. These identified compounds were subsequently uploaded to the PubChem database (https://pubchem.ncbi.nlm.nih.gov/) to obtain their canonical Simplified Molecular Input Line Entry System (SMILES) molecular structures. Furthermore, the chemical formulas of these identified compounds were inputted into the Swiss Target Prediction database (http://www.swisstargetprediction.ch/) for predictive analysis.

### Enrichment analysis and network construction

We conducted an intersection analysis of Chaga-specific targets with markers characteristic of distinct cellular subpopulations to identify shared targets. Subsequently, an enrichment analysis of these intersected genes was performed utilizing the Search Tool for Retrieving Gene/Protein Interactions (STRING) database. This analysis focused on the identification of enriched genes and proteins within the Kyoto Encyclopedia of Genomes (KEGG) Pathway database. Pathways exhibiting q-values significantly below 0.05 were earmarked for detailed examination. This entailed selecting genes with notable regulatory pathway attributes and screening the gene pathway network of principal target genes. The resultant data were then integrated into Cytoscape to construct a comprehensive drug-component-target-pathway network.

### Animals and experiment design

The study was conducted in strict accordance with the ethical guidelines provided by the Ethical Review Committee for Animal Experiments of Shanxi Provincial People’s Hospital, and protocol approval was 2023-245. Male C57BL/6 mice, aged 8 weeks and weighing 20–30 g, were sourced from the Key Laboratory of Nephrology and Laboratory Animal Center of Shanxi Province. These Specific Pathogen-Free (SPF) mice were housed in a controlled, pathogen-free environment with free access to food and water and subjected to a consistent 12-hour light/dark cycle, ensuring uniform environmental conditions for all study participants. Following a week-long acclimatization, the establishment of a murine model of renal fibrosis commenced. This was facilitated through an intraperitoneal injection of 250 mg/kg folic acid (FA; Sigma-Aldrich, Catalog #F7876) dissolved in 0.3 M sodium bicarbonate solution. The control group received equivalent volumes of the sodium bicarbonate solution only. Afterward, the Chaga group was treated with Chaga extract via oral gavage, at a dosage of 300 mg/kg body weight, starting 1 day after FA administration and continuing for 14 days. To investigate the time-dependent effects of Chaga on FA-induced renal fibrosis, mice were segmented into cohorts treated for 3, 7, and 14 days. Kidney tissues were collected for subsequent analysis at the end of each treatment duration, with three mice included per group at every time point. All mice kidneys were subjected to bulk sequencing, and only kidneys from mice in the 14-day group were used for single-cell sequencing.

### Isolation of single cell from murine kidney

The mice were first anesthetized using isoflurane and then perfused with ice-cold 1x phosphate buffered saline (PBS) through the left ventricle. Kidney tissue was then excised and finally cut into small pieces of approximately one cubic millimetre. These tissue sections were then placed in Petri dishes for enzymatic dissociation. The dissociation process involved two stages: initial treatment with GEXSCOPE Tissue Dissociation Solution for 15 min at 37°C, followed by further digestion with 40 µm filtered PBS. Post-digestion, cellular debris was separated from viable cells utilizing a 40-micron Falcon sterile filter. The filtered cell suspension was centrifuged and resuspended in HyClone PBS. GEXSCOPE Erythrocyte Lysis Buffer (Singleron) was used to remove erythrocytes. Cells were subsequently quantified and viability assessed using a TC20 automated cell counter (Bio-Rad). Using these methods, we successfully generated single-cell suspensions with a viability of over 90%.

### Single-cell library generation and sequencing

For the preparation of single-cell libraries, isolated kidney cells were first resuspended in Hank’s Balanced Salt Solution (HBSS). Ensuring cell viability, these cells were then processed using chromium microfluidic chips, utilizing 3′ chemistry (Gel Bead Kit v.3), and were barcoded employing a 10x Chromium Controller (10x Genomics). This step was critical for the subsequent identification and analysis of individual cells. The RNA extracted from these barcoded cells underwent reverse transcription to synthesize complementary DNA (cDNA). Following this, sequencing libraries were constructed using reagents supplied in the Chromium Single Cell 3′ v.3 Reagent Kit (10x Genomics), adhering strictly to the guidelines provided by the manufacturer.

### ScRNA-seq dataset pre-treatment

For our scRNA-seq data, initial processing involved generating raw unique molecular index (UMI) counts per gene per cell using CellRanger, referencing mm10. Quality control measures included the exclusion of genes expressed in less than 3 cells, cells with gene counts below 200, and potential doublets indicated by gene counts exceeding 5,500. Cells with over 30% mitochondrial gene expression, indicative of injury or apoptosis, were also removed, with a higher threshold applied due to the known mitochondrial dynamics in kidney. Normalization of raw gene UMI counts was done to counts per 10,000, followed by log transformation using Seurat. We identified the top 2000 variable genes via Seurat’s FindVariableFeatures with the vst method. Integration of these samples used Seurat’s IntegrateData with 30 dimensions from canonical correlation analysis (CCAs) and 2000 anchors from FindIntegrationAnchors. This integration was vital for uniform gene expression representation in subsequent analyses.

### Dimensionality reduction and clustering analysis

Principal component analysis (PCA) was employed on the scaled dataset, utilizing the RunPCA function with a parameter of 30 principal components (PCs). Subsequently, the first 15 PCs were selected for clustering purposes. The construction of the K-nearest neighbors (KNN) graph, essential for identifying similar cell types, was executed using the FindNeighbors function. Clustering of the cells was then achieved through the FindClusters function, with a resolution parameter set at 0.6. For visual representation of the clustering results, Uniform Manifold Approximation and Projection (UMAP) coordinates were calculated (using the RunUMAP function, dimensions 1–15). This step facilitated the intuitive visualization of the cellular clusters. To discern the differential gene expression across clusters, we applied the Wilcoxon rank-sum test. Marker genes for each cluster were then determined based on a log2 fold change greater than 0.25 and an adjusted p-value below 0.05, as identified using the FindAllMarkers function. Finally, the most highly expressed genes within each cluster were utilized to ascertain the specific cell types, ensuring a robust and precise cell type annotation.

### Bulk RNA-seq library preparation, sequencing and quality control

In this study, total RNA was employed as the starting material for the construction of sequencing libraries using the NEBNext Ultra RNA Library Prep Kit for Illumina, strictly according to the manufacturer’s protocol (Catalog #: E7530L, NEB United States). Indexing was integrated for sequence attribution. mRNA was isolated via poly-T oligo-attached magnetic beads and fragmented under specific ionic and thermal conditions. cDNA synthesis was performed in a two-step process using random hexamer primers and M-MuLV Reverse Transcriptase, followed by DNA Polymerase I and RNase H for second-strand synthesis. The subsequent end-repair process yielded blunt-ended cDNA, which was then adenylated and ligated to adaptors. Fragment size selection targeted a range of 370–420 bp, with AMPure XP system purification. cDNA amplification involved USER Enzyme and Phusion High-Fidelity DNA polymerase, and the final product was evaluated using the Agilent 5,400 system and quantified by QPCR to a precise concentration. Quality control was paramount, involving the conversion of raw fluorescence image files to FASTQ format. Fastp was employed to remove adapter sequences, discard reads with uncertain bases above a 10% threshold, and exclude reads where low-quality bases exceeded 50%. This rigorous QC process ensured the retention of only high-fidelity reads for downstream analysis. Sequencing was performed on an Illumina platform with a paired-end 150 strategy by Novogene Bioinformatics Technology Co. Ltd, optimized for the required data output and library concentration. Differential expression was analyzed with DESeq2, yielding log2 fold changes along with p-values and adjusted p-values. Visualization of bulk RNA-seq data was performed using the ggplot2 package.

### Cell trajectory reconstruction using Monocle2 pseudotime analysis

To understand the developmental progression of renal cells, we employed Monocle2, a sophisticated computational method that orders single cells along pseudotime trajectories. Through this approach, we were able to reconstruct biological processes by examining the sequential changes in gene expression that cells undergo during their dynamic developmental journey. Initially, we utilized the Seurat framework to process and normalize our single-cell RNA sequencing data, which was then converted into the CellDataSet (CDS) format required for Monocle2 analysis. Subsequently, by applying the differentialGeneTest function, we successfully identified genes that showed significant association with different cellular states (q-value <0.01). Based on these findings, we proceeded with dimensionality reduction and trajectory construction to better understand the cellular dynamics. Furthermore, we implemented reversed graph embedding to determine the precise ordering of cells along the trajectory, thereby enabling us to effectively map the progression of cellular states during renal fibrosis development. Finally, to visualize these complex cellular evolutionary paths, we employed the plot_cell_trajectory function, where we used different colors to represent distinct cell states and highlighted branch points to indicate critical fate decisions.

### Cell–cell communications analysis

Cell-to-cell communication was analyzed using the CellChat R package, which interprets complex ligand-receptor interactions across distinct cell types. Normalized gene expression matrices, along with primary cell type classifications from Control, Renal fibrosis, and Chaga groups, served as the foundational data inputs for CellChat. The ‘mergeCellChat' function was instrumental in integrating and comparing interaction networks. Quantitative assessment of intercellular communication diversity involved calculating the variance in ligand-receptor pairings among the aforementioned groups. For cell types characterized by significant variability, the ‘subsetCellChat’ function permitted a granular examination of subtypes. The analytic process culminated with the identification of ligand-receptor pairs uniquely expressed within each group. Suboptimal pairs, as indicated by insufficient mean interaction values in CellChat’s output, were systematically excluded to ensure the integrity of the interaction data.

### Public spatial transcriptomics analysis

The public spatial transcriptomics data and the corresponding single-cell data were derived from GSE154107 and GSE151658, respectively. Firstly, we used our identified marker gene for the re-annotation of the single cell sequencing data in GSE151658. After this re-annotation, we proceeded to delineate anchors that facilitated a linkage between the comprehensively integrated single-cell entities and the spatial transcriptomics dataset (Hao et al., 2024).

### Kidney histology

Kidney samples were fixed in 10% formalin and subsequently embedded in paraffin. Sections of 4 µm thickness were prepared from these formalin-fixed, paraffin-embedded (FFPE) samples. We employed Hematoxylin and Eosin (H&E) staining, as well as Masson’s trichrome staining, to evaluate histological changes. These changes included tubular atrophy, dilation of tubular lumens, infiltration of interstitial immune cells, and collagen deposition. Imaging was performed using a Leica DM4000B microscope at both ×200 magnifications. The degree of tubular necrosis was scored using a semiquantitative scale (Muratsu et al., 2022): 0 = normal kidney; 1 = minimal necrosis (5% involvement); 2 = mild necrosis (5%–25% involvement); 3 = moderate necrosis (25%–50% involvement); 4 = severe necrosis (50%–75% involvement); and 5 = most severe necrosis (75% involvement). Semiquantitative analysis of Masson staining was calculated based on the percentage of collagen-positive area.

### Statistical analysis

For all data, normality was first assessed using the Shapiro-Wilk test. For normally distributed data, results are expressed as mean ± standard deviation (x ± s), and one-way analysis of variance (ANOVA) is used to assess statistical differences among the three groups. For non-normally distributed data, results are presented as median (interquartile range) P50 (P25, P75), and the Kruskal–Wallis test is employed to evaluate statistical differences among the three groups. Multiple comparisons between groups are performed using the Bonferroni correction method. The following symbols are used to indicate different levels of statistical significance: *p < 0.05, **p < 0.01, ***p < 0.001.

## Results

### Identification of compounds and compound-targets in chaga

In this study, UPLC-MS was employed for a systematic and comprehensive analysis of the chemical constituents of Chaga. The total ion chromatograms of Chaga, under both positive and negative ion modes, are depicted in Supplementary Figure S1A, B. This technique facilitated the separation and detection of 60 distinct compounds in Chaga, as listed in Supplementary Table S1. Upon inputting the structures of the 60 identified compounds into the Swiss Target Prediction Search Server, a total of 675 related targets were discerned (Supplementary Table S2). The 60 compounds identified, along with the 675 targets elucidated, will be used in the construction of subsequent network.

### HE and masson staining

**FIGURE 1:**
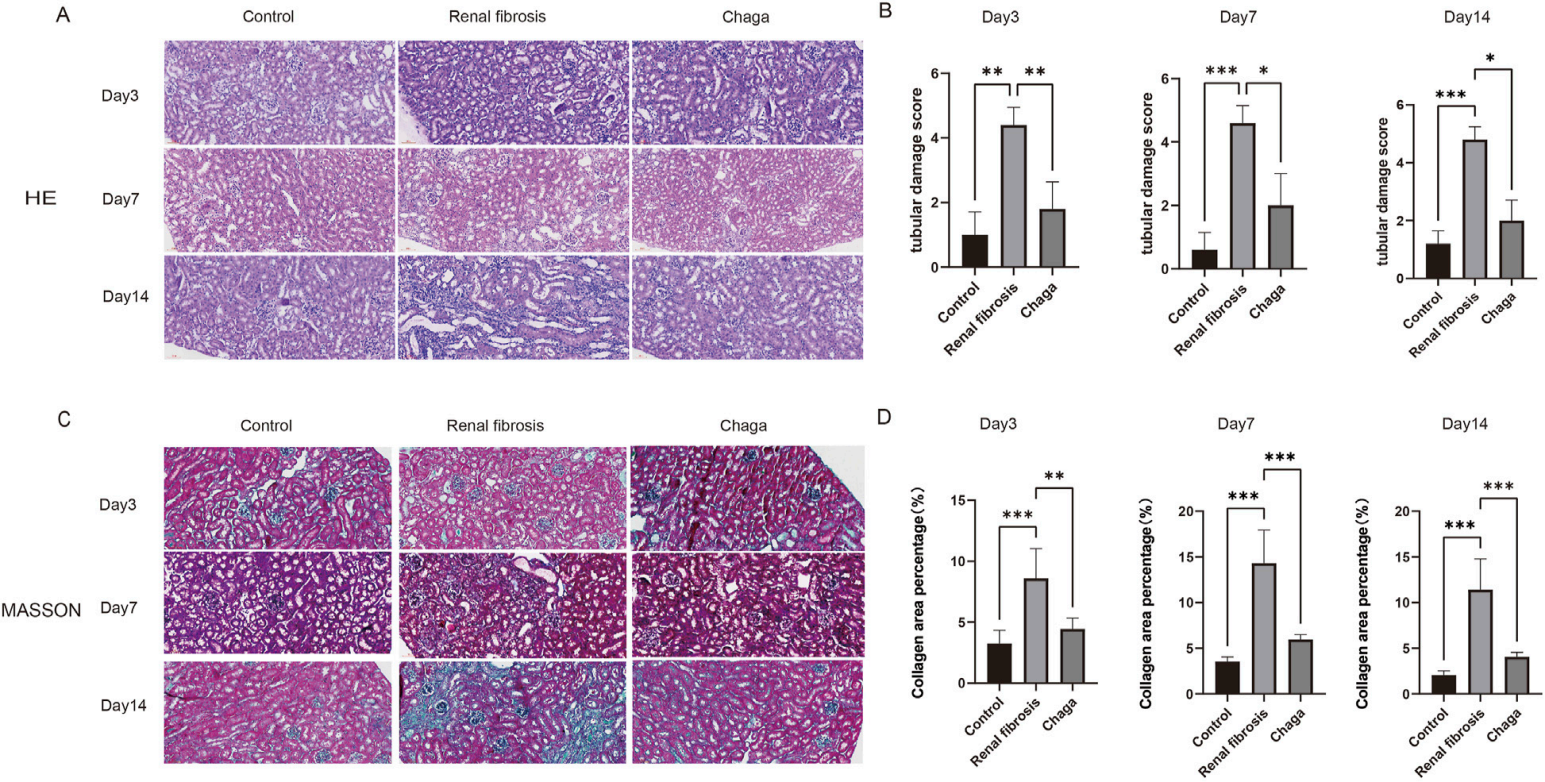
Representative histological sections (magnification: ×200). **(A)** HE staining of kidney sections from control, renal fibrosis, and Chaga-treated groups at days 3, 7, and 14. **(B)** The tubular damage scores, presented as median (P25, P75), were as follows: at day 3, control [1.00 (1.00, 1.00)], renal fibrosis [4.00 (4.00, 5.00)], and Chaga-treated [2.00 (1.00, 2.00)]; at day 7, control [1.00 (0.00, 1.00)], renal fibrosis [5.00 (4.00, 5.00)], and Chaga-treated [2.00 (1.00, 3.00)]; at day 14, control [1.00 (1.00, 1.00)], renal fibrosis [5.00 (5.00, 5.00)], and Chaga-treated [2.00 (2.00, 2.00)]. **(C)** Masson’s trichrome staining of kidney sections from control, renal fibrosis, and Chaga-treated groups at days 3, 7, and 14. **(D)** The Masson’s trichrome staining quantification, the area percentages of fibrosis, presented as mean ± SD, were as follows: at day 3, control (3.26 ± 0.96), renal fibrosis (8.59 ± 2.20), and Chaga-treated (4.44 ± 0.81); at day 7, control (3.54 ± 0.46), renal fibrosis (14.31 ± 3.25), and Chaga-treated (5.95 ± 0.50); at day 14, control (2.05 ± 0.43), renal fibrosis (11.40 ± 3.01), and Chaga-treated (4.06 ± 0.45). *P < 0.05, **P < 0.01, ***P < 0.001.

**FIGURE 2:**
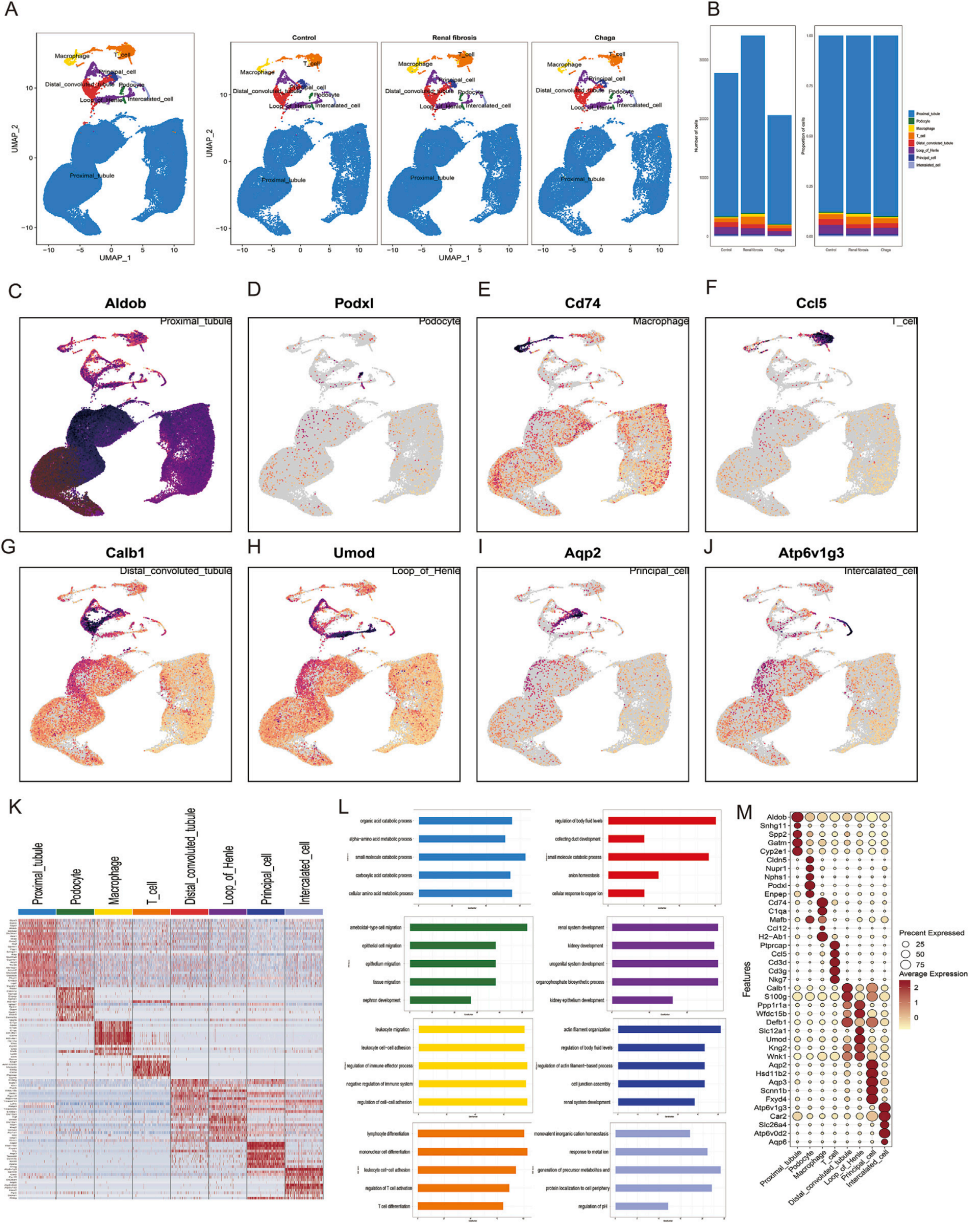
Characterization of Cellular Heterogeneity in Mouse Kidney Cells via Single-Cell Transcriptomics **(A)** Integration of samples subjected to various treatments (Control, Renal Fibrosis, Chaga) into a unified dataset, followed by clustering utilizing uniform manifold approximation and projection (UMAP) **(B)** Enumeration and distribution of discerned cell types across each experimental condition **(C–J)** UMAP projections depicting the expression patterns of selected hallmark genes corresponding to each cell type **(K)** Heatmap representation of genes exhibiting differential expression across eight identified cellular clusters **(L)** Gene Ontology (GO) analysis associated with these clusters **(M)** Dot plot visualization highlighting the expression levels of characteristic marker genes within the identified cell types.

In HE staining of the kidney, we saw clear signs of renal injury in the renal fibrosis group on day 14 of the experiment, including tubular dilatation, epithelial cell flattening and inflammatory cell infiltration (Figure 1A). Renal tubular damage scores showed that control [1.00 (1.00, 1.00)], renal fibrosis [5.00 (5.00, 5.00)], and Chaga-treated [2.00 (2.00, 2.00)], respectively (p < 0.05), whereas the Chaga treatment significantly reduced renal damage in terms of pathological manifestation. This pattern was consistently mirrored in the pathological examinations conducted at days 3 and 7 of the experimental timeline (Figure 1B). Parallel observations were noted in Masson’s trichrome staining across the time points of days 3, 7, and 14. A conspicuous augmentation in the blue staining indicative of collagen was noted within the renal interstitial areas of the fibrosis group when compared to controls (p < 0.05). Conversely, Chaga treatment ameliorated collagen accumulation (p < 0.05), suggesting an efficacious role of Chaga in attenuating the progression of renal fibrosis (Figures 2C, D).

### Single-cell transcriptomic profiling suggested differential gene expression patterns across cell types

We performed scRNA-seq of the kidneys from these mice as described in the methods and illustrated in the previous workflow chart. In total, 82,496 single-cell transcriptomes were generated after quality control and filtering. 30 separate cell clusters were identified after pooling all the samples together (Supplementary Figure S2). The 30 clusters were classified into 8 cell types and annotated based on cell-specific marker genes reported in previous kidney single-cell data, and they were consistent across all three groups (Wu et al., 2019; Zambrano et al., 2022; Conway et al., 2020) (Figure 2A). To ascertain the alterations in predominant cell types following renal fibrosis and subsequent Chaga treatment, we conducted a comparative analysis of cell populations across three groups. It was observed that proximal tubular (PT) cells constituted the principal component in each group. Additionally, a notably higher proportion of macrophages and T cells was detected in the renal fibrosis group compared to the control and Chaga-treated groups. (Figure 2B). Represent marker genes of each cluster are shown in Figures 2C–J. The heatmap (Figure 2K) and dotplot (Figure 2M) show the expression patterns of key genes across various renal cell populations, including proximal tubule cells, podocytes, macrophages, T-cells, distal convoluted tubule cells, loop of Henle cells, principal cells, and intercalated cells. The GO analysis, performed on the combined data from multiple groups, yielded insights into the biological processes that are active across the mixed cell populations (Figure 2L). The analysis revealed a broad spectrum of processes, including metabolic pathways and cellular responses to various stimuli. Clusters of immune cell migration and cell-cell adhesion were observed, which may reflect a range of cell states or responses within the heterogeneous sample.

### Subpopulation dynamics and pathway enrichment analysis in PT cells

**FIGURE 3:**
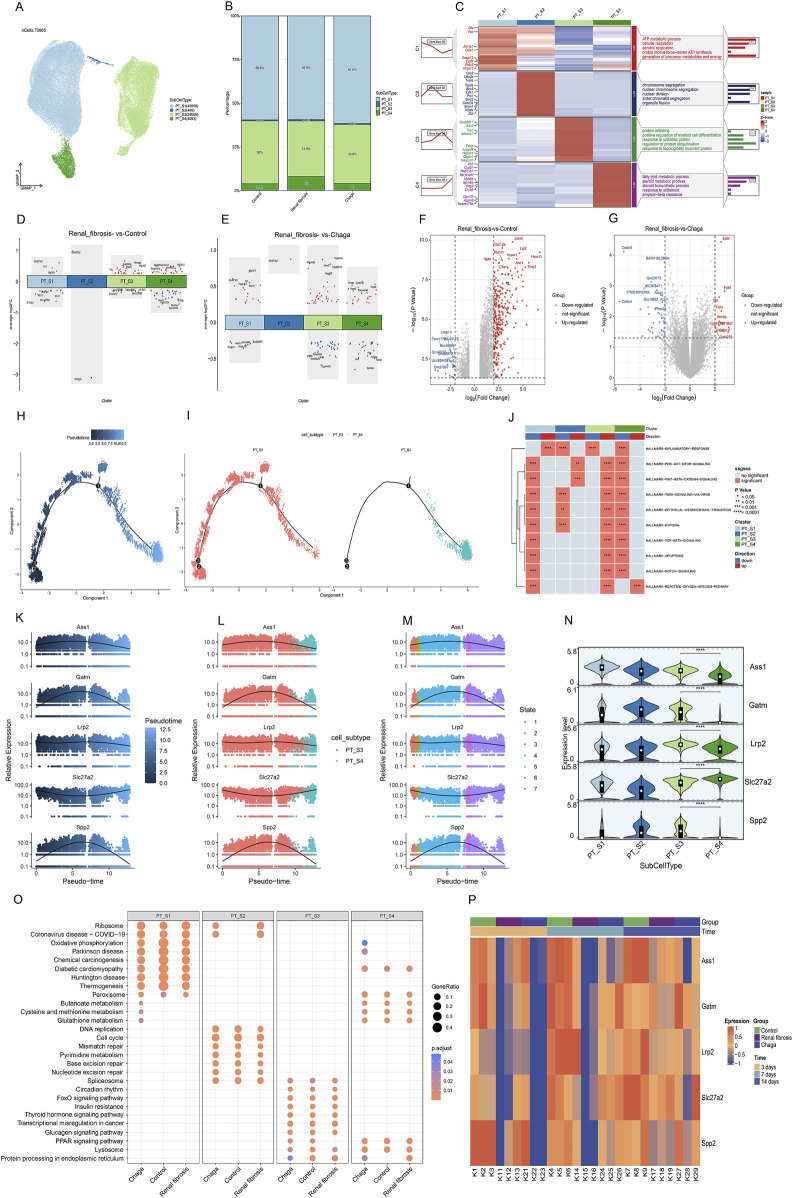
PT cells responses to Chaga treatment in renal fibrosis through single-cell and bulk sequencing **(A)** UMAP plot visualizing subclusters of PT cells **(B)** Bar graphs depicting the alterations in the percentage of PT cell subpopulations between groups **(C)** Heatmap of PT cell subpopulations and pathway enrichment results for each subgroup **(D)** Volcano plot showing differentially expressed genes of the 4 subclusters between Renal fibrosis and Control group **(E)** Volcano plot showing differentially expressed genes of the 4 subclusters between Renal fibrosis and Chaga group **(F–G)** Volcano plots showing the differentially expressed genes between the renal fibrosis group and the control group, as well as between the renal fibrosis group and the Chaga-treated group, based on Bulk sequencing data **(H–I)** Monocle trajectories of PT cells coloured by different pseudotimes. Each dot represents a cell **(J)** Heatmap from the GSEA revealing significant pathways across four PT cell clusters **(K–M)** Gene(Ass1, Galm, Lrp2, Slc27a2, and Spp2) expression across pseudotime within cell subtypes and different states **(N)** violin plots indicating expression levels across four PT cell subtypes **(O)** Dotplot visualization of pathway enrichment analysis, comparing the impact of Chaga treatment, Control, and Renal fibrosis conditions across PT cell subtypes **(P)** Heatmap displaying time-course gene expression changes for five genes across control, Renal fibrosis, and chaga treatment groups.

Given that PT cells emerged as the predominant cell type in our experiment, and aligned with prior research that underscores heterogeneity in the response of diverse PT cells under pathological conditions (Hinze et al., 2022; Wu et al., 2022). We further conducted a subpopulation annotation of PT cells, delineating them based on the specificity of their markers (Liao et al., 2020; Lu et al., 2021) (Figure 3A). Our findings intriguingly revealed a notably diminished prevalence of the S3 subgroup in the renal fibrosis group compared to both the control and Chaga treatment groups. Conversely, the prevalence of the S4 subgroup was significantly elevated in the renal fibrosis group when contrasted with the control and Chaga treatment cohorts (Figure 3B). The S3 subpopulation was notably enriched in pathways related to “protein refolding”, “response to unfolded proteins”, and “regulation of ubiquitin-protein ligase activity in the mitotic cell cycle”. Meanwhile, the S4 subpopulation showed a distinct enrichment in pathways pertaining to “fatty acid metabolism”, “steroid metabolism”, “response to steroids”, and “amyloid-β clearance” (Figure 3C). The enrichment pathway of S3, S4 may be relevant for regulation after cell damage or in disease states, and we next analyzed the differences between conditions for the four subpopulations (Figures 3D, E). We found that the S3 and S4 subpopulations exhibited a significantly higher number of differential genes compared to S1 and S2, both in comparisons between renal fibrosis and control groups, as well as between renal fibrosis and Chaga treatment groups. This suggested that S3 and S4 might be particularly responsive to Chaga in mitigating renal fibrosis, evident in both disease progression and treatment phases. We also performed differential gene analysis on the Bulk sequencing data as well, and finally we identified 273 genes that were overlappingly upregulated (meaning that these 273 genes were upregulated in the renal fibrosis group, but the Chaga treatment reversed the change (Figures 3F, G). The next GO enrichment analysis revealed that primarily in these genes were mainly enriched in the biology of the leukocytes in the immune response (Supplementary Figure S3A).

Next, we conducted a pseudo-temporal analysis to re-evaluate the differences between S3 and S4 subpopulations, discovering that S3 is distributed throughout the entire time series. Meanwhile, S4 appears exclusively in the later time points (Figures 3H, I). Further, we scrutinized the top 100 genes (Supplementary Figure S3B) in the time series and successfully identified 5 genes with notably different expression levels within S3 and S4 (Figures 3K–N). we also identified these 60 genes that were almost all upregulated in the renal fibrosis group in the bulk sequencing data (Supplementary Figure S3C). According to Figure 3O, Supplementary Figure S4 was distinguished by its enrichment in oxidative phosphorylation, whereas the pathways of the three subgroups, S1, S2, S3, were similar under different conditions. Additionally, the regulation of S4 in oxidative phosphorylation by Chaga was similarly found in the Reatome enrichment analyses (Supplementary Figure S3D–F). In the Bulk sequencing data, we also found a clear temporal effect in that these 5 genes were less expressed in the renal fibrosis group on day 3, day 7, and highly expressed on day 14, showing a clear temporal effect (Figure 3P).

### Role of chaga on macrophage subpopulations in mice with renal fibrosis

**FIGURE 4:**
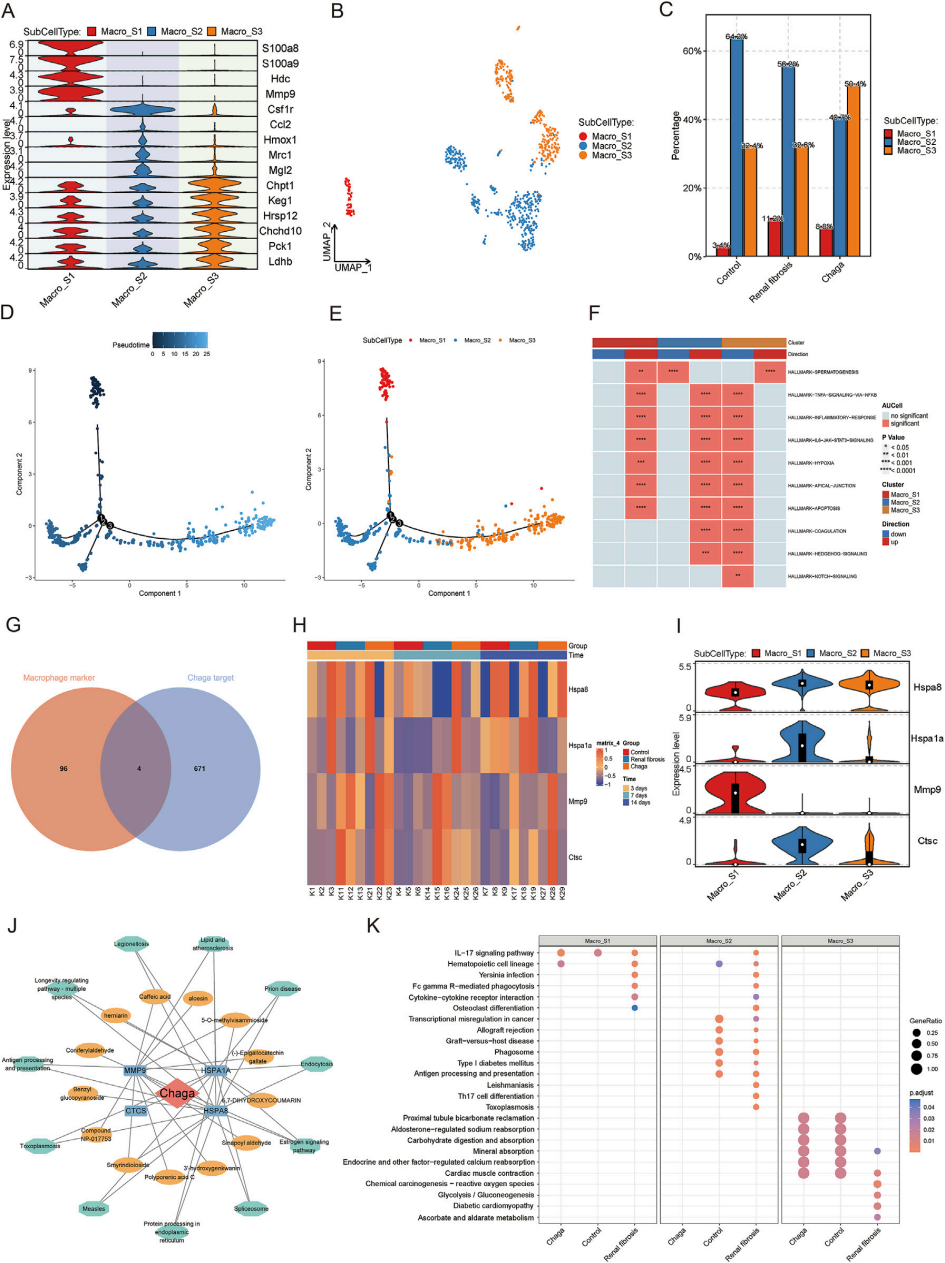
The effects of chaga treatment on macrophage subpopulations in renal fibrosis based on single-cell sequencing, bulk sequencing, and network pharmacology analysis **(A)** Heatmap of macrophage subpopulation groupings of the maker gene **(B)** UMAP plot of macrophage subpopulations **(C)** Bar graph of variability in the percentage of macrophage subpopulations between different groups **(D)** Demonstrating macrophages differentiation trajectories using pseudo-temporal color mapping techniques **(E)** Cell trajectory visualization with color-coded display of sub-cell types (Macro_S1, Macro_S2, Macro_S3) **(F)** Heatmap from the GSEA revealing significant pathways across three macrophage subclusters **(G)** Overlapping targets of macrophage marker genes and Chaga targets **(H)** Heatmap of candidate gene expression patterns in Bulk sequencing data **(I)** Violin plots of candidate gene expression in single-cell sequencing data **(J)** Network pharmacology analysis depicting compound-target-pathways network for Chaga **(K)** Dotplot visualization of pathway enrichment analysis, comparing the impact of Chaga treatment, Control, and Renal fibrosis conditions across macrophage subtypes.

Macrophages are key cellular components of the kidney and are essential for maintaining tissue homeostasis and ensuring a rapid response to kidney injury (Chen et al., 2019; Privratsky et al., 2023; Wen et al., 2019). Our prior analysis has demonstrated a notable augmentation in macrophage populations in the context of renal fibrosis pathogenesis, meanwhile, the administration of Chaga appeared to mitigate this excessive macrophage aggregation. As shown in Figures 4A, B and Supplementary Figure S4A, we next categorized macrophages into three sub-populations (Macro_S1, Macro_S2, and Macro_S3) based on their specific markers. S100a8 and S100a9, which are considered as marker genes for M1-type macrophages, are highly expressed by the S1 subpopulation, and we therefore consider the S1 subpopulation to be the pro-inflammatory M1 phenotype. Hmox1, Mrc1 (also known as Cd206) and MgI2 are markers of the M2 type of macrophage. We therefore suggest that Macro_S2 is an anti-inflammatory, tissue repair oriented M2 phenotype. By analyzing the proportions of each subpopulation in different conditions, we found that M1 was significantly increased in the renal fibrosis group compared to the healthy group, but Chaga intervention similarly reduced the aggregation of M1 phenotype. Then for M2 macrophages, the proportion was significantly lower in the renal fibrosis group than in the healthy group, but not higher in the Chaga group than in the renal fibrosis group (Figure 4C). We then used Monocle2 to infer the differentiation trajectories of macrophage subpopulations S1, S2, and S3 from single-cell sequencing data (Figure 4D). Figure 4E and Supplementary Figure S4B described the successive differentiation process from S1 to S3 may correspond to the transition from the initial immune response to later stages associated with tissue repair. Thus, these panels reveal a dynamic differentiation of macrophages from M1-like to M2-like states in the pathogenesis of renal fibrosis. According to Figure 4F, the inflammatory response signals we screened that signals (TNF-signaling, inflammatory-response, IL6-Jak-Stat3-signaling, and apoptosis pathways) that may be driven by macrophages and thus lead to cause kidney injury, are significantly upregulated in S1,S2, which are at the early stage of the trajectory, and significantly downregulated in S3, which are all at the late stage of the trajectory. In addition, we took the intersection of the top 100 maker genes of macrophages obtained by single-cell sequencing and 675 drug potential targets of Chaga obtained previously, and finally identified four intersecting genes (Hspa8, Hspa1a, Mmp9, and Ctsc) (Figure 4G). We extended to investigate the sensitivity of these four candidate genes to Chaga across multiple time points in Bulk sequencing data. We found that Hspa1a was significantly increased in the renal fibrosis group compared to the control group at 14 days, whereas Chaga could reduce its expression to control group levels. The same expression pattern was seen for Mmp9 and Ctsc at the time point of 7th day (Figure 4H). In single-cell sequencing data we also found that Hspa1a and Ctsc were highly expressed in S2, while Mmp9 was highly expressed in S1 (Figure 4I).

A network was constructed to study the effect of Chaga for the treatment of renal fibrosis targeting macrophage with multiple components, targets, and pathways (Figure 4J). This network intuitively reflected the potential substance basis and mechanism of action. In the enrichment analysis, we discerned a discernible decrement in the enrichment of certain inflammatory and immunological pathways within the S2 sub-group of the ‘Chaga' cohort when juxtaposed with the ‘Renal fibrosis' group. Pertinently, the pathways encompassing ‘IL-17 signaling pathway,' ‘Cytokine-cytokine receptor interaction,' ‘Antigen processing and presentation,' and ‘Th17 cell differentiation' were implicated. A parallel diminution was also manifest in the S1 sub-group, notably within the ‘IL-17 signaling pathway' and ‘Antigen processing and presentation' pathways (Figure 4K).

### Modulatory effects of chaga on T cell subsets in renal fibrosis with insights into immunological pathways and molecular mechanisms

**FIGURE 5:**
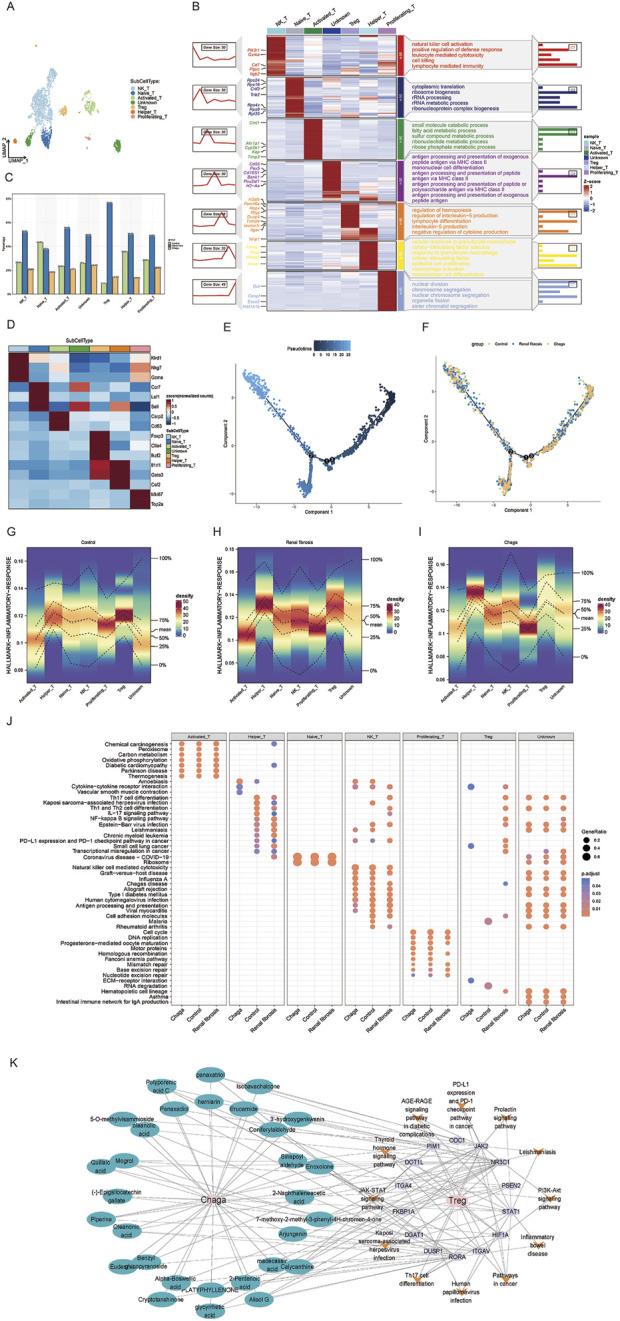
Single-cell sequencing reveals the response of T-cell subsets to Chaga treatment during renal fibrosis **(A)** UMAP clustering of T cell subtypes from single-cell RNA-seq data **(B)** Heatmap showing gene expression and pathway enrichment results in T cell subgroups **(C)** Bar chart of T cell subtype frequencies across samples **(D)** Marker gene expression heatmap for T cell subtypes **(E)** Pseudo-temporal trajectory of T cell differentiation **(F)** Developmental trajectories of T Cells from different groups **(G–I)** Density plots depicting the distribution of T cell subsets in the inflammatory response pathway of control, renal fibrosis and Chaga-treatment group **(J)** Dotplot visualization of pathway enrichment analysis, comparing the impact of Chaga treatment, Control, and Renal fibrosis conditions across T Cells subtypes **(K)** Network pharmacology diagram detailing the interactions between Chaga compounds, their target genes, and the consequent effects on T cell pathway signaling.

T cells are integral in renal fibrosis pathogenesis, serving as a crucial cellular entity that modulates the inflammatory milieu precipitating tissue fibrosis, thus emerging as potential targets for therapeutic intervention (Gao et al., 2021; Liu et al., 2021; Tapmeier et al., 2010). Our prior investigations delineated a pronounced increase in T-cell populations in renal fibrosis, which was substantially mitigated following Chaga administration. Acknowledging the heterogeneity of T cell subpopulations, disparate immunological landscapes emerged Chaga treatment as compared to renal fibrosis. Subsequent stratification of T cells revealed six subsets, natural killer T cell (NKT), Naive T, Activated T, regulatory T cell (Treg), Helper T, and Proliferating T, as well as an undefined group, delineated by specific markers (Figures 5A, D). Excluding Naive T cells, a consistent expression pattern was observed across the other subsets (Figure 5C). Gene clustering unveiled seven expression trends, signifying diverse biological functionalities. Notably, NK T cells exhibited upregulation in genes associated with cytotoxicity and immune defense, whereas Tregs displayed enrichment in genes governing hematopoiesis and immunosuppression, highlighting the intricate pathophysiology of renal fibrosis involving NK T cell-mediated cytotoxicity and Treg-driven homeostatic repair mechanisms (Figure 5B). We then analyzed the dynamic progression of T cell subsets in the control, renal fibrosis, and Chaga-treated groups by means of pseudotime trajectories. For NK T cells, an active role in the immune response was already present from early in the time series. In contrast, Treg cells showed a trajectory of involvement in the later stages of the response, consistent with their role in immunomodulation and repair of homeostasis. We also found that the T cell subsets in the Chaga group were located early in the time series, and it is possible that Chaga modulates immune function to achieve remission from the early stages of the renal fibrosis process (Figures 5E, F; Supplementary Figure S5).

We also conducted an evaluation of the enrichment of T cells in various inflammatory response pathways under different conditions. Notably, our findings revealed that NK T and Treg cells exhibited the high level of enrichment in the context of renal fibrosis. With Chaga intervention, there was a significant reduction in the enrichment of NK T cells and Treg in inflammatory signaling pathways (Figures 5G–I). Furthermore, enrichment analysis of T cell subsets additionally indicated that Chaga treatment attenuated the activity of NK T cells and Treg in the IL-17 and NF-kappa B signaling pathways (Figure 5J). Consequently, these results suggest that Chaga can modulate the functional activity of T cell subsets, thereby influencing immune function and ultimately exerting an anti-fibrotic effect. A network was also constructed to study the effect of Chaga for the treatment of renal fibrosis targeting Treg with multiple components, targets and pathways, thereby uncovering potential small molecular compounds that modulate Treg cell differentiation via Th17 cell pathways, revealing the intricate molecular mechanisms involved (Figure 5K).

### Cellular communication in renal fibrosis and chaga intervention studies

**FIGURE 6:**
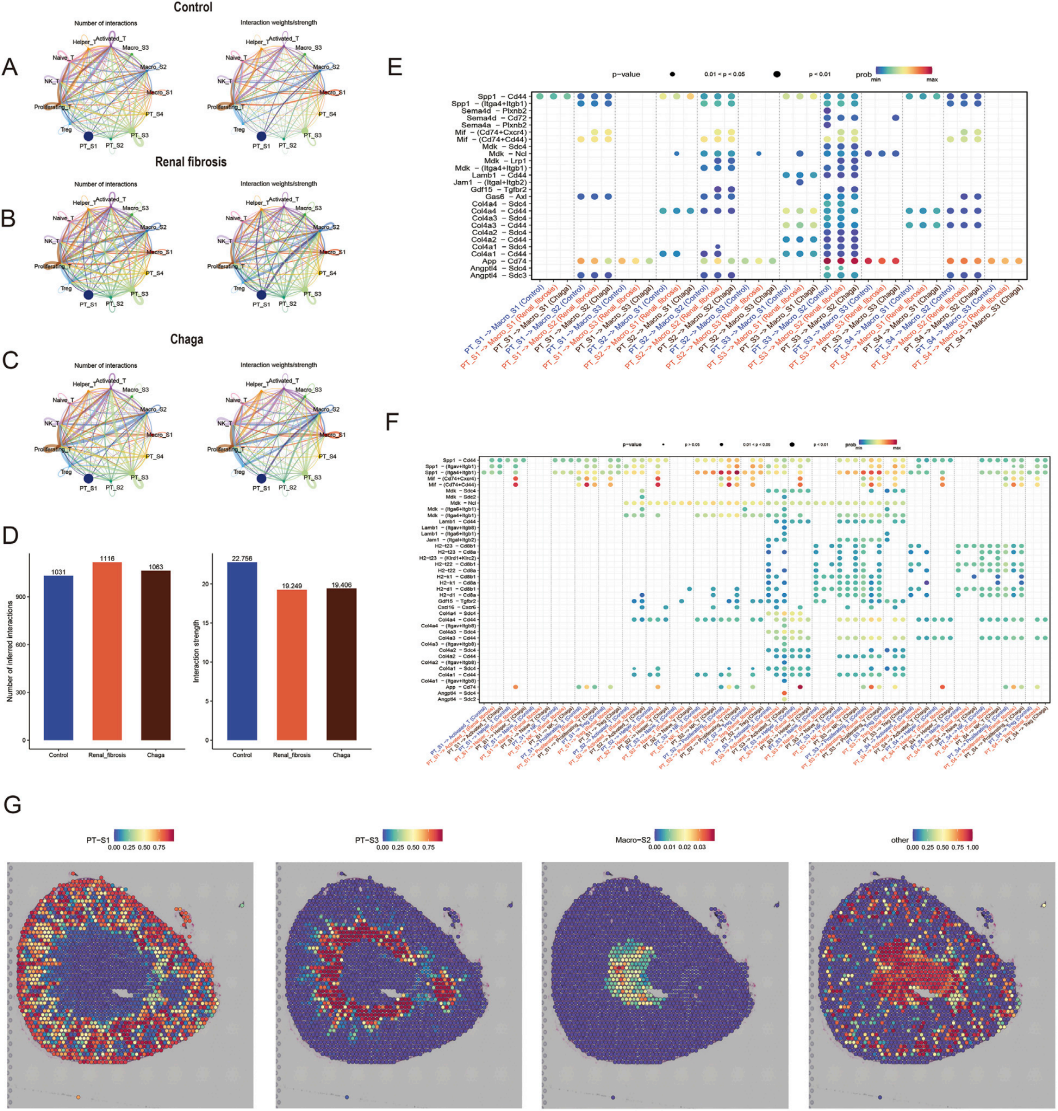
Chaga rewired intercellular crosstalk in renal fibrosis **(A–C)** Chord plot of the total number of cell-cell interactions between the control, renal fibrosis and Chaga groups, coloured based on each cell type and degree of thickness **(D)** the number of crosstalk inferred interactions (left) and the strength of interactions (right) between cells in each group. The bobble plot shows significant upregulated ligand receptor pairs between sender and receiver cell **(E)** between PTs and macrophages **(F)** between PTs and T Cells **(G)** Integration of spatial transcriptomics with scRNA-seq identifies the proximity of PT_S3 and Macro_S2.

To explore potential changes in cell-to-cell communication during renal fibrosis and following Chaga intervention, we analyzed interaction patterns across the three experimental groups (Figures 6A–C). The numerical data visualized in Figure 6D indicated that the renal fibrosis group had 1,116 total interactions compared to 1,031 in the control group, while the average interaction strength was 19.249 in the fibrosis group compared to 22.758 in controls. Following Chaga treatment, we observed 1,063 total interactions with an average strength of 19.406. These observations suggested possible alterations in intercellular communication patterns that may relate to disease progression and treatment effects, though these represented descriptive trends rather than statistically validated differences.

We next compared the interactions where renal tubular epithelial cells, acting as ligands, affected T cells and macrophages. Notably, we identified that ligand-receptor pairs such as Lamb1 - Cd44, Col4a - Cd44, and Col4a - Sdc4 were predominantly expressed in the PT_S3 (Figures 6E, F). This finding corroborates our earlier hypothesis, suggesting that PT_S3 might represent a subgroup of renal tubular epithelial cells particularly sensitive to Chaga intervention. Additionally, we integrated and deconvoluted published spatial transcriptomics data with accompanying single-cell transcriptomics data (Janosevic et al., 2021), enabling a reannotation of our findings. Interestingly, this integrative approach revealed that the Macro_S2 and PT_S3 subgroups occupy closer spatial positions within renal tissue compared to other subgroups of renal tubular epithelial cells (Figure 6G). This spatial proximity between Macro_S2, an anti-inflammatory and tissue repair oriented M2 phenotype, and PT_S3, may yield significant insights into the cellular dynamics and interactions within the kidney. This understanding is particularly crucial in the context of renal fibrosis and Chaga intervention, highlighting a potential nexus point for therapeutic exploration.

## Discussion

The rising prevalence of CKD has become a substantial global health burden. Irrespective of its etiology, renal fibrosis is renal fibrosis is recognised as a universal pathological feature and an end stage in the progression of chronic kidney disease (Huang et al., 2023; Li et al., 2022). The identification of an expanding array of pharmacological interventions, including Renin-Angiotensin System (RAS) blockers, Sodium-Glucose Co-Transporter 2 (SGLT2) inhibitors, vasopressin receptor 2 antagonists, and nonsteroidal mineralocorticoid receptor antagonists, which have been demonstrated to decelerate the advancement of CKD, represents a significant breakthrough (Zhong et al., 2015; Yang et al., 2022). Additionally, a plethora of herbal formulations, encompassing both singular herb extracts and their combinations, have been empirically validated to mitigate renal fibrosis (Guo et al., 2017; Zhong et al., 2019). Rigorously conducted experimental investigations have elucidated their antifibrotic mechanisms and efficacies. In this study, cellular mapping of mouse kidneys after administration of Chaga for the treatment of renal fibrosis using single-cell RNA sequencing constructs was employed. This approach was complemented by UPLC-MS analysis and network pharmacology to identify pharmacological targets and elucidate the molecular basis of the renoprotective properties of the molecular constituents of Chaga against renal fibrosis to promote renoprotection.

Research extensively demonstrated the critical involvement of PT cells in the pathogenesis of early and late DKD (Gilbert, 2017; Zeni et al., 2017), while RAS blockade, SGLT2 inhibitors are all using PT cells as the main target of action (Sinha and Agarwal, 2019; Wang et al., 2023; Zhu et al., 2023). Therefore, we firstly focused on PT cells. In our study, we identified four PT subpopulations based on their specific expression of gene marker as well as cellular function. Sdc4 was highly expressed in PT_S3 subpopulation *versus* the other subpopulations, and Sdc4 knockdown was shown to lead to a reduction in extracellular transglutaminase-2 and to prevent tubulointerstitial fibrosis (Scarpellini et al., 2014). Recent studies have shown that aging biomarkers are downregulated in TXNIP knockout mice and that these effects can lead to attenuation of renal fibrosis and renal impairment (He et al., 2021). In pseudotime analysis of PT_S3 and PT_S4 subgroups, it was observed that the expression of Slc7a2 and Gatm in PT_S3 is significantly higher than in the PT_S4 subgroup. A recent study reported that Slc27a2 knockout DKD susceptible mice exhibit normalized renal function, reduced proteinuria, improved renal histopathology and longer lifespan (Khan et al., 2020). Heterozygous GATM mutations have been observed to cause mitochondrial abnormalities and renal fibrosis caused by inflammasome activation (Reichold et al., 2018). Additionally, our investigation revealed that gene expression profiles between PT_S3 and PT_S4 subgroups disclosed a greater number of differentially expressed genes within the renal fibrosis cohort in comparison to both the normal and Chaga-treated groups. This suggests that gene alterations in PT_S3 and PT_S4 subgroups are significant during the progression from normal renal function to renal fibrosis and following Chaga treatment, highlighting their potential as key therapeutic targets for renal fibrosis. Notably, GO enrichment analysis of these pivotal genes primarily identified immune response and immunoregulatory pathways (Supplementary Figure S3A), underscoring the critical role of immune mechanisms in both the pathogenesis and treatment of renal fibrosis.

Macrophages are central to both monitoring immune integrity and maintaining kidney homeostasis, exhibiting a dynamic response spectrum to renal damage contingent on the insult’s nature and duration (Nikolic-Paterson et al., 2011; Calle and Hotter, 2020; Huen and Cantley, 2017). These cells manifest diverse phenotypes ranging from M1 pro-inflammatory, crucial for infection eradication but potentially exacerbating renal harm, to M2 anti-inflammatory, which embody a restorative phenotype aiding in injury resolution (Kumar, 2018; Hu et al., 2021). This versatility underscores their complex influence on renal pathophysiology and recovery processes. In our study, we classified macrophages into 3 subpopulations based on existing studies, where Macro_S1 showed M1-type macrophage characteristics and Macro_S2 showed M2-type macrophage characteristics. Among the changes in the proportion of macrophages under different conditions, we found that the therapeutic effect of Chaga may be achieved by decreasing the aggregation of M1-type pro-inflammatory macrophages rather than increasing the anti-inflammatory and repairing effect of M2-type macrophages (Figure 4C). We also found that the Fc gamma R-mediated phagocytosis pathway of Macro_S1 was downregulated in the Chaga-treated group compared to the renal fibrosis group, implying that Chaga may attenuate the M1-type macrophage-mediated inflammatory response and ameliorate renal injury (Figure 4K). Through the integration of network pharmacological analysis of Chaga’s molecular components and macrophage markers, we identified four common genes: Hspa8, Hspa1a, Mmp9, and Ctsc, with MMP9 notably overexpressed in the Macro_S1 subtype. MMP9 has been implicated in the orchestration of pro-inflammatory macrophage recruitment to the renal milieu during the course of experimental crescentic glomerulonephritis, signifying its critical role in the inflammatory cascade and pathogenesis of renal disorders (Kluger et al., 2013). Our investigation suggested that Chaga may mitigate renal fibrosis by modulating Mmp9 expression *via* its distinct molecular constituents, underscoring a potential therapeutic mechanism of action against renal fibrotic processes.

Increasing evidence has highlighted the regulatory role of T cells in renal fibrosis, however, the contribution of T cell subsets to this pathological condition remains insufficiently elucidated (Dong et al., 2016; Wen et al., 2020). Consequently, leveraging single cell sequencing technology, we have discerned the functional paradigms of disparate T cell subpopulations within the milieu of renal fibrosis and subsequent Chaga treatment. Paramount among our findings is the pronounced augmentation of inflammatory responses in Tregs and NKTs in the renal fibrosis group compared to controls (Figures 5G, H). This potentiation of inflammation was effectively attenuated in Chaga-treated specimens, which intimates the potential modulatory capacity of Chaga treatment on inflammation engendered by renal fibrosis. Furthermore, in our pathway enrichment analyses, Chaga therapy was found to diminish the activity of NKT cells and Tregs within the IL-17 and NF-kappa B signaling pathways, postulating that Chaga may temper T cell-mediated immune functions, thereby mitigating the inflammatory-driven progression of renal fibrosis. Additionally, our construction of a network pharmacology network—encompassing drug-components-targets-pathways—has illuminated that constituents of Chaga may interact with Tregs and ultimately exert immunomodulatory effects through DUSP1 in Th17 cell differentiation, which is consistent with some previous studies (Sheng et al., 2019; Shi et al., 2023; Park et al., 2022). Ergo, further studies are imperative to ascertain the salutary contributions of T cell subsets to renal fibrosis, with the aim of advancing efficacious therapeutic interventions.

We also conducted an in-depth cell-to-cell communication crosstalk analysis revealing pivotal interactions between proximal tubular (PT) cells and immune cells, namely, macrophages and T cells, which underpin inflammatory injury and tissue repair mechanisms within the renal fibrotic process. We identified potential ligand-receptor interactions within the renal microenvironment modulated by folate-induced renal fibrosis and subsequent treatment with Chaga. Notably, PT cells demonstrated significant interactions with macrophages and T cells in the domains of cell adhesion, chemokine receptor pairs, indicating a robust recruitment and activation of immune cells facilitated by intercellular engagements, specifically through Spp1 — Cd44, Mif — (Cd74+Cd44), Gdf15 — Tgfrb2, and Angptl4 — Sdc4 (Zhou et al., 2023; Kong et al., 2022). Moreover, our study discerned enhanced cellular communication between the Chaga-sensitive PT_S3 subpopulation and the anti-inflammatory reparative Macro_S2 subpopulation, more so than with other groups. Intriguingly, upon re-annotating published spatial transcriptomics data, we discovered a closer spatial proximity within the kidney between the PT_S3 and Macro_S2 subgroups (Figure 6G). This proximity not only corroborated the significance of these subpopulations in the pathology of renal fibrosis but also suggested that their spatial relationship may elucidate the observed therapeutic efficacy of Chaga treatment. Our study elucidated the intricacies of cellular heterogeneity and targeted communication within renal fibrotic subpopulations at the single-cell and spatial levels, uncovering potential targets for precision medicine and personalized therapy in kidney fibrosis.

The main limitations of this study include: (1) the small number of samples for single-cell and bulk transcriptome analysis, which may affect the statistical power of our findings; (2) the lack of experimental validation for the computational predictions from network pharmacology analysis; (3) the need for more comprehensive clinical sample validation. Although we utilized multiple approaches including network pharmacology, public spatial transcriptome, single-cell sequencing data, and pathology experiments to support our findings, these results need to be further confirmed by larger single-cell studies, mechanistic experiments, and clinical sample validations.

## Conclusion

Our investigation explored the complex intercellular dynamics in the context of folate-induced renal fibrosis and its potential modulation by Chaga treatment. Through single-cell sequencing analysis, we observed interactions among endothelial cells, proximal tubular (PT) cells, macrophages, and T Cells, providing insights into the cellular crosstalk patterns associated with inflammatory and fibrotic responses in CKD. The integration of our findings suggested that Chaga treatment may influence cellular interactions and tissue repair processes in renal fibrosis. While our observations in this mouse model indicate potential therapeutic effects of Chaga, further experimental validation is needed to establish its clinical application. These preliminary findings may provide new directions for investigating novel interventions in the management of CKD.

## Data Availability

The original contributions presented in the study are publicly available. This data can be found here: https://doi.org/10.6084/m9.figshare.26885686.v1.

## References

[B1] Calle P. Hotter G. (2020). Macrophage phenotype and fibrosis in diabetic nephropathy. Int. J. Mol. Sci. 21, 2806. 10.3390/ijms21082806 32316547 PMC7215738

[B2] Chen T. Cao Q. Wang Y. Harris D. C. H. (2019). M2 macrophages in kidney disease: biology, therapies, and perspectives. Kidney Int. 95, 760–773. 10.1016/j.kint.2018.10.041 30827512

[B3] Conway B. R. O’Sullivan E. D. Cairns C. O’Sullivan J. Simpson D. J. Salzano A. (2020). Kidney single-cell atlas reveals myeloid heterogeneity in progression and regression of kidney disease. J. Am. Soc. Nephrol. 31, 2833–2854. 10.1681/ASN.2020060806 32978267 PMC7790206

[B4] Dong Y. Yang M. Zhang J. Peng X. Cheng J. Cui T. (2016). Depletion of CD8+ T cells exacerbates CD4+ T cell-induced monocyte-to-fibroblast transition in renal fibrosis. J. Immunol. 196, 1874–1881. 10.4049/jimmunol.1501232 26773152

[B5] Fordjour E. Manful C. F. Javed R. Galagedara L. W. Cuss C. W. Cheema M. (2023). Chaga mushroom: a super-fungus with countless facets and untapped potential. Front. Pharmacol. 14, 1273786. 10.3389/fphar.2023.1273786 38116085 PMC10728660

[B6] Gao M. Wang J. Zang J. An Y. Dong Y. (2021). The mechanism of CD8+ T cells for reducing myofibroblasts accumulation during renal fibrosis. Biomolecules 11, 990. 10.3390/biom11070990 34356613 PMC8301885

[B7] Gilbert R. E. (2017). Proximal tubulopathy: prime mover and key therapeutic target in diabetic kidney disease. Diabetes 66, 791–800. 10.2337/db16-0796 28325740

[B8] Guo L. Luo S. Du Z. Zhou M. Li P. Fu Y. (2017). Targeted delivery of celastrol to mesangial cells is effective against mesangioproliferative glomerulonephritis. Nat. Commun. 8, 878. 10.1038/s41467-017-00834-8 29026082 PMC5638829

[B9] Hao Y. Stuart T. Kowalski M. H. Choudhary S. Hoffman P. Hartman A. (2024). Dictionary learning for integrative, multimodal and scalable single-cell analysis. Nat. Biotechnol. 42, 293–304. 10.1038/s41587-023-01767-y 37231261 PMC10928517

[B10] He Q. Li Y. Zhang W. Chen J. Deng W. Liu Q. (2021). Role and mechanism of TXNIP in ageing-related renal fibrosis. Mech. Ageing Dev. 196, 111475. 10.1016/j.mad.2021.111475 33781783

[B11] Hinze C. Kocks C. Leiz J. Karaiskos N. Boltengagen A. Cao S. (2022). Single-cell transcriptomics reveals common epithelial response patterns in human acute kidney injury. Genome Med. 14, 103. 10.1186/s13073-022-01108-9 36085050 PMC9462075

[B12] Hu Q. Lyon C. J. Fletcher J. K. Tang W. Wan M. Hu T. Y. (2021). Extracellular vesicle activities regulating macrophage- and tissue-mediated injury and repair responses. Acta Pharm. Sin. B 11, 1493–1512. 10.1016/j.apsb.2020.12.014 34221864 PMC8245807

[B13] Huang R. Fu P. Ma L. (2023). Kidney fibrosis: from mechanisms to therapeutic medicines. Signal Transduct. Target Ther. 8, 129. 10.1038/s41392-023-01379-7 36932062 PMC10023808

[B14] Huen S. C. Cantley L. G. (2017). Macrophages in renal injury and repair. Annu. Rev. Physiol. 79, 449–469. 10.1146/annurev-physiol-022516-034219 28192060

[B15] Janosevic D. Myslinski J. McCarthy T. W. Zollman A. Syed F. Xuei X. (2021). The orchestrated cellular and molecular responses of the kidney to endotoxin define a precise sepsis timeline. Elife 10, e62270. 10.7554/eLife.62270 33448928 PMC7810465

[B16] Jovic D. Liang X. Zeng H. Lin L. Xu F. Luo Y. (2022). Single-cell RNA sequencing technologies and applications: a brief overview. Clin. Transl. Med. 12, e694. 10.1002/ctm2.694 35352511 PMC8964935

[B17] Khan S. Gaivin R. Abramovich C. Boylan M. Calles J. Schelling J. R. (2020). Fatty acid transport protein-2 regulates glycemic control and diabetic kidney disease progression. JCI Insight 5, e136845. 10.1172/jci.insight.136845 32614804 PMC7455077

[B18] Kluger M. A. Zahner G. Paust H.-J. Schaper M. Magnus T. Panzer U. (2013). Leukocyte-derived MMP9 is crucial for the recruitment of proinflammatory macrophages in experimental glomerulonephritis. Kidney Int. 83, 865–877. 10.1038/ki.2012.483 23344471

[B19] Kong Y.-Z. Chen Q. Lan H.-Y. (2022). Macrophage migration inhibitory factor (MIF) as a stress molecule in renal inflammation. Int. J. Mol. Sci. 23, 4908. 10.3390/ijms23094908 35563296 PMC9102975

[B20] Kou R.-W. Han R. Gao Y.-Q. Li D. Yin X. Gao J.-M. (2021). Anti-neuroinflammatory polyoxygenated lanostanoids from Chaga mushroom Inonotus obliquus. Phytochemistry 184, 112647. 10.1016/j.phytochem.2020.112647 33434790

[B21] Kumar S. (2018). Cellular and molecular pathways of renal repair after acute kidney injury. Kidney Int. 93, 27–40. 10.1016/j.kint.2017.07.030 29291820

[B22] Li L. Fu H. Liu Y. (2022). The fibrogenic niche in kidney fibrosis: components and mechanisms. Nat. Rev. Nephrol. 18, 545–557. 10.1038/s41581-022-00590-z 35788561

[B23] Liang Z. Tang Z. Zhu C. Li F. Chen S. Han X. (2024). Intestinal CXCR6+ ILC3s migrate to the kidney and exacerbate renal fibrosis via IL-23 receptor signaling enhanced by PD-1 expression. Immunity 57, 1306–1323.e8. 10.1016/j.immuni.2024.05.004 38815582 PMC11539045

[B24] Liao J. Yu Z. Chen Y. Bao M. Zou C. Zhang H. (2020). Single-cell RNA sequencing of human kidney. Sci. Data 7, 4. 10.1038/s41597-019-0351-8 31896769 PMC6940381

[B25] Liu B. Jiang J. Liang H. Xiao P. Lai X. Nie J. (2021). Natural killer T cell/IL-4 signaling promotes bone marrow-derived fibroblast activation and M2 macrophage-to-myofibroblast transition in renal fibrosis. Int. Immunopharmacol. 98, 107907. 10.1016/j.intimp.2021.107907 34243040

[B26] Lu Y.-A. Liao C.-T. Raybould R. Talabani B. Grigorieva I. Szomolay B. (2021). Single-nucleus RNA sequencing identifies new classes of proximal tubular epithelial cells in kidney fibrosis. J. Am. Soc. Nephrol. 32, 2501–2516. 10.1681/ASN.2020081143 34155061 PMC8722798

[B27] Mencke R. Olauson H. Hillebrands J.-L. (2017). Effects of Klotho on fibrosis and cancer: a renal focus on mechanisms and therapeutic strategies. Adv. Drug Deliv. Rev. 121, 85–100. 10.1016/j.addr.2017.07.009 28709936

[B28] Muratsu J. Sanada F. Koibuchi N. Shibata K. Katsuragi N. Ikebe S. (2022). Blocking periostin prevented development of inflammation in rhabdomyolysis-induced acute kidney injury mice model. Cells 11, 3388. 10.3390/cells11213388 36359784 PMC9658410

[B29] Nikolic-Paterson D. J. Wang S. Lan H. Y. (2011). Macrophages promote renal fibrosis through direct and indirect mechanisms. Kidney Int. Suppl. 4, 34–38. 10.1038/kisup.2014.7 PMC4536961 26312148

[B30] Papalexi E. Satija R. (2018). Single-cell RNA sequencing to explore immune cell heterogeneity. Nat. Rev. Immunol. 18, 35–45. 10.1038/nri.2017.76 28787399

[B31] Park S. Lee H. Lee J. Lee S. Cho S. Huh H. (2022). RNA-seq profiling of tubulointerstitial tissue reveals a potential therapeutic role of dual anti-phosphatase 1 in glomerulonephritis. J. Cell Mol. Med. 26, 3364–3377. 10.1111/jcmm.17340 35488446 PMC9189340

[B32] Privratsky J. R. Ide S. Chen Y. Kitai H. Ren J. Fradin H. (2023). A macrophage-endothelial immunoregulatory axis ameliorates septic acute kidney injury. Kidney Int. 103, 514–528. 10.1016/j.kint.2022.10.008 36334787 PMC9974788

[B33] Reichold M. Klootwijk E. D. Reinders J. Otto E. A. Milani M. Broeker C. (2018). Glycine amidinotransferase (GATM), renal fanconi syndrome, and kidney failure. J. Am. Soc. Nephrol. 29, 1849–1858. 10.1681/ASN.2017111179 29654216 PMC6050927

[B34] Ruiz-Ortega M. Lamas S. Ortiz A. (2022). Antifibrotic agents for the management of CKD: a review. Am. J. Kidney Dis. 80, 251–263. 10.1053/j.ajkd.2021.11.010 34999158

[B35] Scarpellini A. Huang L. Burhan I. Schroeder N. Funck M. Johnson T. S. (2014). Syndecan-4 knockout leads to reduced extracellular transglutaminase-2 and protects against tubulointerstitial fibrosis. J. Am. Soc. Nephrol. 25, 1013–1027. 10.1681/ASN.2013050563 24357671 PMC4005302

[B36] Sheng J. Li H. Dai Q. Lu C. Xu M. Zhang J. (2019). DUSP1 recuses diabetic nephropathy via repressing JNK-Mff-mitochondrial fission pathways. J. Cell Physiol. 234, 3043–3057. 10.1002/jcp.27124 30191967

[B37] Shi L. Zha H. Pan Z. Wang J. Xia Y. Li H. (2023). DUSP1 protects against ischemic acute kidney injury through stabilizing mtDNA via interaction with JNK. Cell Death Dis. 14, 724. 10.1038/s41419-023-06247-4 37935658 PMC10630453

[B38] Sinha A. D. Agarwal R. (2019). Clinical pharmacology of antihypertensive therapy for the treatment of hypertension in CKD. Clin. J. Am. Soc. Nephrol. 14, 757–764. 10.2215/CJN.04330418 30425103 PMC6500954

[B39] Tang P.M.-K. Nikolic-Paterson D. J. Lan H.-Y. (2019). Macrophages: versatile players in renal inflammation and fibrosis. Nat. Rev. Nephrol. 15, 144–158. 10.1038/s41581-019-0110-2 30692665

[B40] Tapmeier T. T. Fearn A. Brown K. Chowdhury P. Sacks S. H. Sheerin N. S. (2010). Pivotal role of CD4+ T cells in renal fibrosis following ureteric obstruction. Kidney Int. 78, 351–362. 10.1038/ki.2010.177 20555323

[B41] Wang Z. Zhai J. Zhang T. He L. Ma S. Zuo Q. (2023). Canagliflozin ameliorates epithelial-mesenchymal transition in high-salt diet-induced hypertensive renal injury through restoration of sirtuin 3 expression and the reduction of oxidative stress. Biochem. Biophys. Res. Commun. 653, 53–61. 10.1016/j.bbrc.2023.01.084 36857900

[B42] Wen Y. Lu X. Ren J. Privratsky J. R. Yang B. Rudemiller N. P. (2019). KLF4 in macrophages attenuates tnfα-mediated kidney injury and fibrosis. J. Am. Soc. Nephrol. 30, 1925–1938. 10.1681/ASN.2019020111 31337692 PMC6779357

[B43] Wen Y. Rudemiller N. P. Zhang J. Robinette T. Lu X. Ren J. (2020). TNF-α in T lymphocytes attenuates renal injury and fibrosis during nephrotoxic nephritis. Am. J. Physiol. Ren. Physiol. 318, F107-F116–F116. 10.1152/ajprenal.00347.2019 PMC6985827 31736350

[B44] Wu H. Kirita Y. Donnelly E. L. Humphreys B. D. (2019). Advantages of single-nucleus over single-cell RNA sequencing of adult kidney: rare cell types and novel cell states revealed in fibrosis. J. Am. Soc. Nephrol. 30, 23–32. 10.1681/ASN.2018090912 30510133 PMC6317600

[B45] Wu J. Sun Z. Yang S. Fu J. Fan Y. Wang N. (2022). Kidney single-cell transcriptome profile reveals distinct response of proximal tubule cells to SGLT2i and ARB treatment in diabetic mice. Mol. Ther. 30, 1741–1753. 10.1016/j.ymthe.2021.10.013 34678510 PMC9077318

[B46] Yang L. Wang B. Ma L. Fu P. (2022). Traditional Chinese herbs and natural products in hyperuricemia-induced chronic kidney disease. Front. Pharmacol. 13, 971032. 10.3389/fphar.2022.971032 36016570 PMC9395578

[B47] Zambrano S. He L. Kano T. Sun Y. Charrin E. Lal M. (2022). Molecular insights into the early stage of glomerular injury in IgA nephropathy using single-cell RNA sequencing. Kidney Int. 101, 752–765. 10.1016/j.kint.2021.12.011 34968552

[B48] Zeni L. Norden A. G. W. Cancarini G. Unwin R. J. (2017). A more tubulocentric view of diabetic kidney disease. J. Nephrol. 30, 701–717. 10.1007/s40620-017-0423-9 28840540 PMC5698396

[B49] Zhang Y. Liao H. Shen D. Zhang X. Wang J. Zhang X. (2021). Renal protective effects of inonotus obliquus on high-fat diet/streptozotocin-induced diabetic kidney disease rats: biochemical, color Doppler ultrasound and histopathological evidence. Front. Pharmacol. 12, 743931. 10.3389/fphar.2021.743931 35111043 PMC8801815

[B50] Zhong Y. Lee K. Deng Y. Ma Y. Chen Y. Li X. (2019). Arctigenin attenuates diabetic kidney disease through the activation of PP2A in podocytes. Nat. Commun. 10, 4523. 10.1038/s41467-019-12433-w 31586053 PMC6778111

[B51] Zhong Y. Menon M. C. Deng Y. Chen Y. He J. C. (2015). Recent advances in traditional Chinese medicine for kidney disease. Am. J. Kidney Dis. 66, 513–522. 10.1053/j.ajkd.2015.04.013 26015275

[B52] Zhou M. Lu F. Jiang L. Chen C. Chen S. Geng L. (2023). Decoding the intercellular cross-talking between immune cells and renal innate cells in diabetic kidney disease by Bioinformatics. J. Inflamm. Res. 16, 3049–3062. 10.2147/JIR.S409017 37497063 PMC10368133

[B53] Zhu Z. Rosenkranz K. A. T. Kusunoki Y. Li C. Klaus M. Gross O. (2023). Finerenone added to RAS/SGLT2 blockade for CKD in alport syndrome. Results of a randomized controlled trial with Col4a3-/- mice. J. Am. Soc. Nephrol. 34, 1513–1520. 10.1681/ASN.0000000000000186 37428955 PMC10482061

