# Corrigendum: Inonotus obliquus (chaga) ameliorates folic acid-induced renal fibrosis in mice: the crosstalk analysis among PT cells, macrophages and T cells based on single-cell sequencing

**DOI:** 10.3389/fphar.2025.1629322

**Published:** 2025-05-29

**Authors:** Yueling Peng, Yaling Zhang, Rui Wang, Xinyu Wang, Xingwei Liu, Hui Liao, Rongshan Li

**Affiliations:** ^1^ Department of Nephrology, Fifth Hospital of Shanxi Medical University (Shanxi Provincial People’s Hospital), Taiyuan, China; ^2^ Department of Nephrology, Taiyuan Central Hospital, Taiyuan, China; ^3^ Drug Clinical Trial Institution, Shanxi Provincial People’s Hospital (Fifth Hospital of Shanxi Medical University), Taiyuan, China

**Keywords:** renal fibrosis, single-cell RNA sequencing, traditional Chinese medicine, proximal tubular cells, macrophages, t cells

In the published article, there was an error in **Affiliation 1**. Instead of “Department of Nephrology, Shanxi Provincial People’s Hospital (Fifth Hospital of Shanxi Medical University), Taiyuan, China”, it should be “Department of Nephrology, Fifth Hospital of Shanxi Medical University (Shanxi Provincial People’s Hospital), Taiyuan, China”.

The authors apologize for this error and state that this does not change the scientific conclusions of the article in any way. The original article has been updated.

