# Supplementary figures and images for: Inonotus obliquus (chaga) ameliorates folic acid-induced renal fibrosis in mice: the crosstalk analysis among PT cells, macrophages and T cells based on single-cell sequencing
Source: Front Pharmacol. 2025 Mar 14;16:1556739. doi: 10.3389/fphar.2025.1556739 (PMC11949929; doi:10.3389/fphar.2025.1556739)

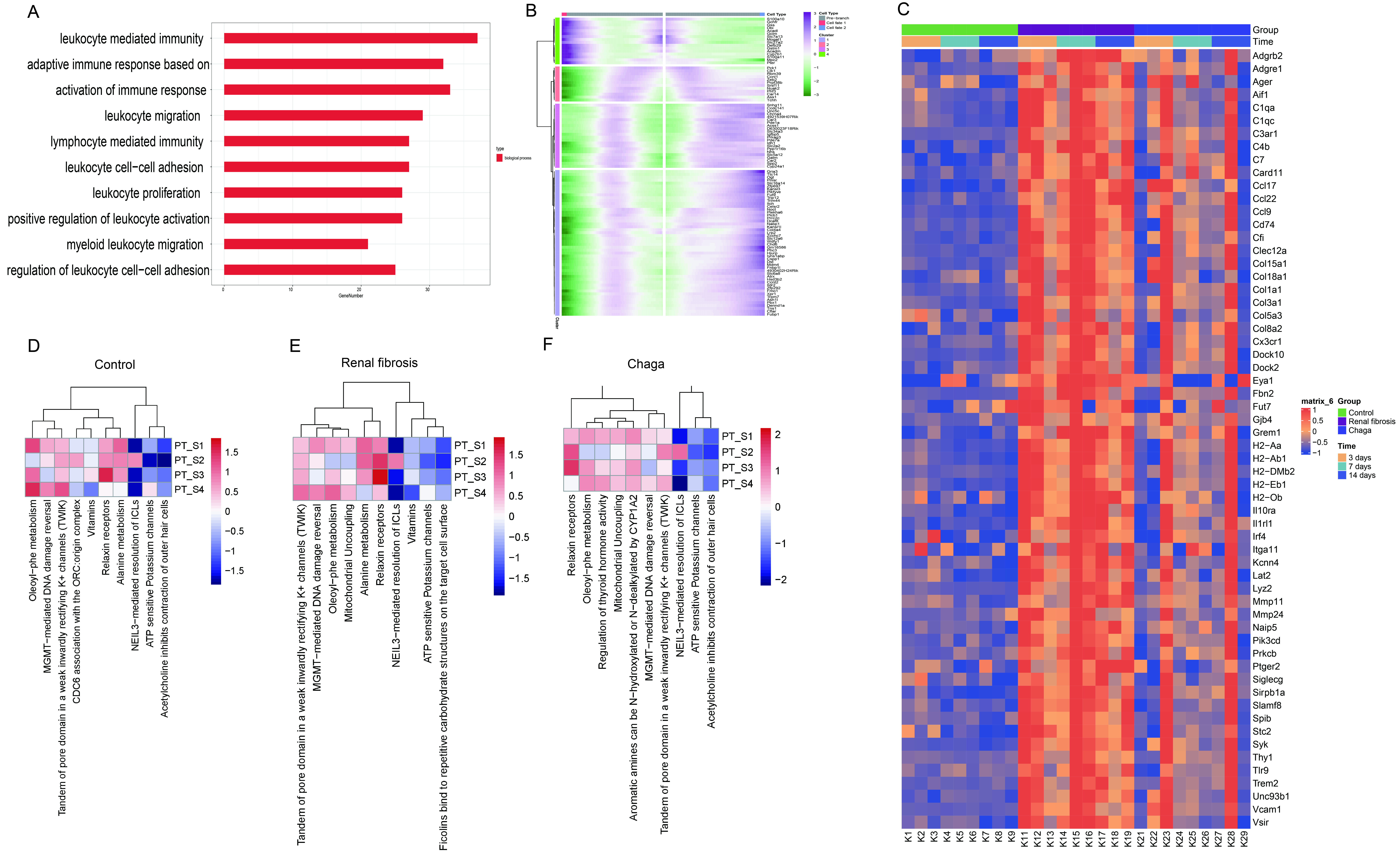

Supplement: Supplementary file 2 [file Image3.jpeg]

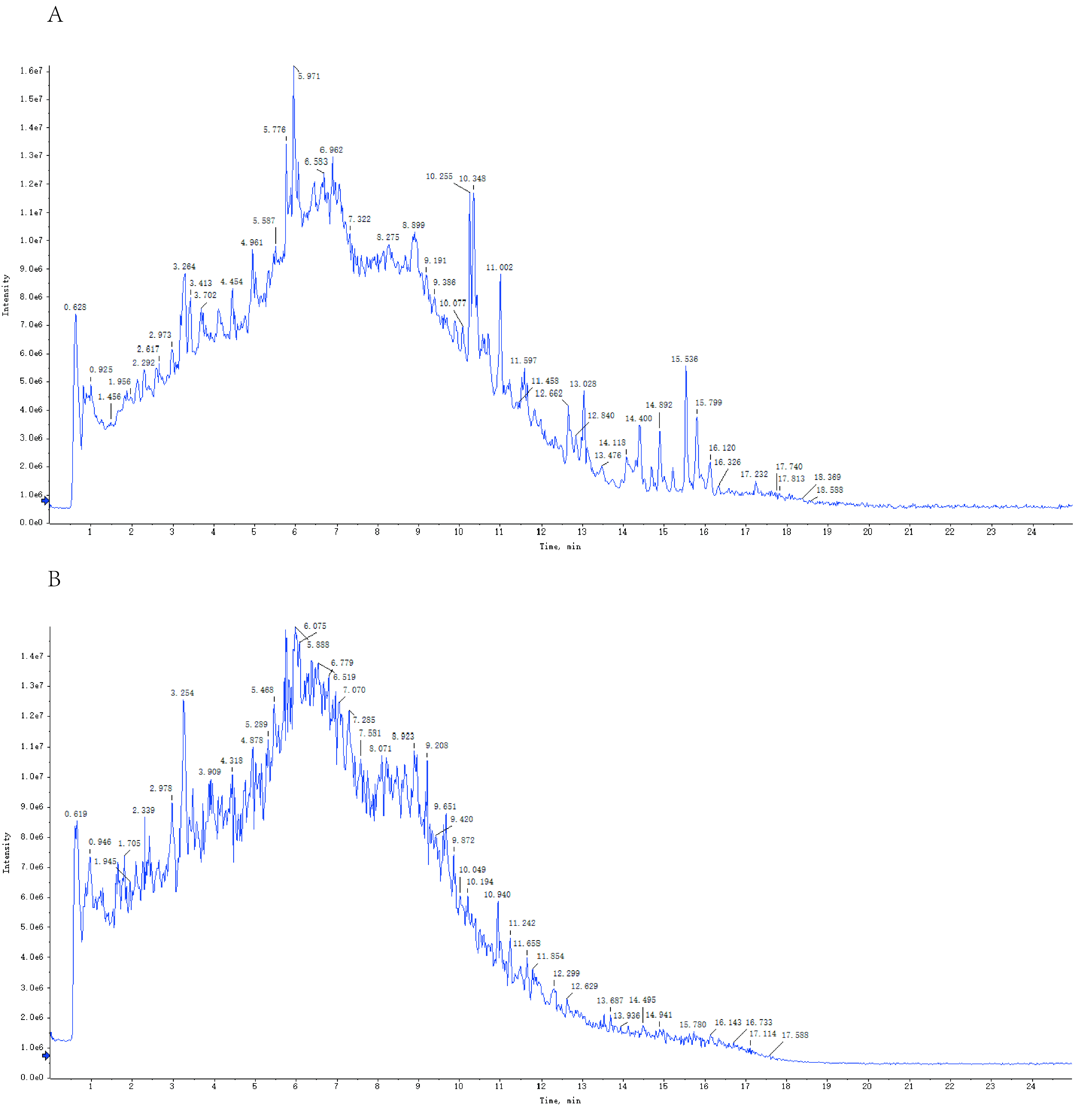

Supplement: Supplementary file 3 [file Image1.jpeg]

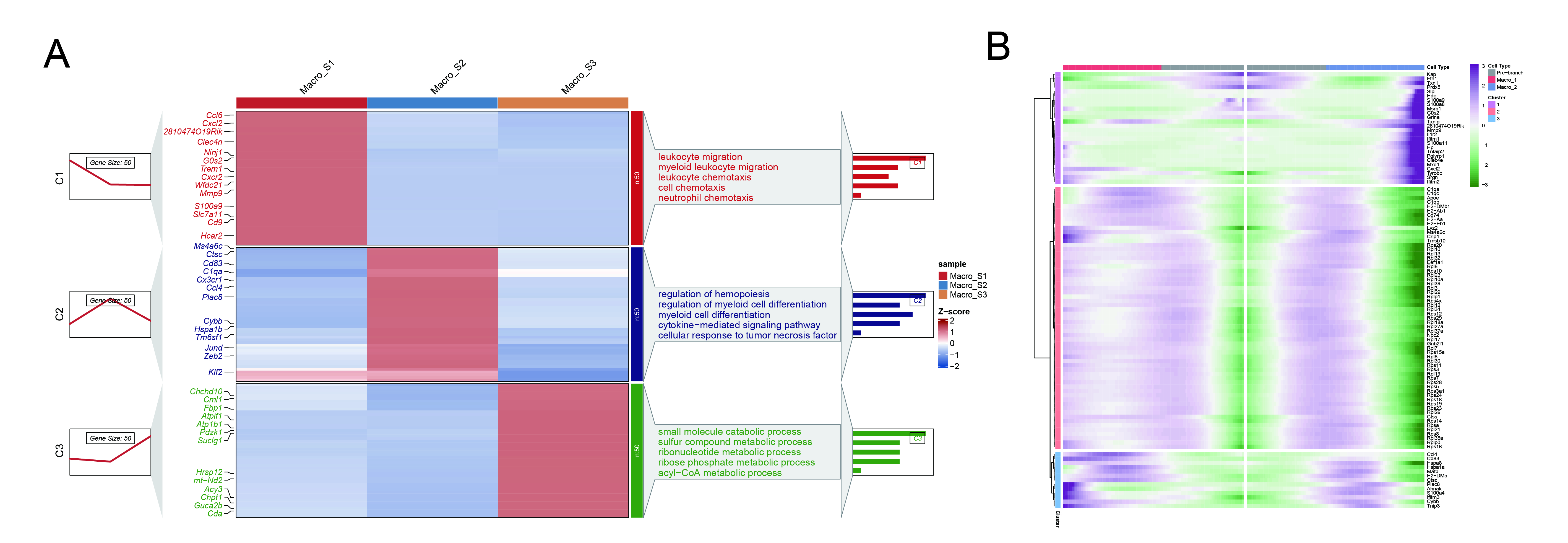

Supplement: Supplementary file 4 [file Image4.jpeg]

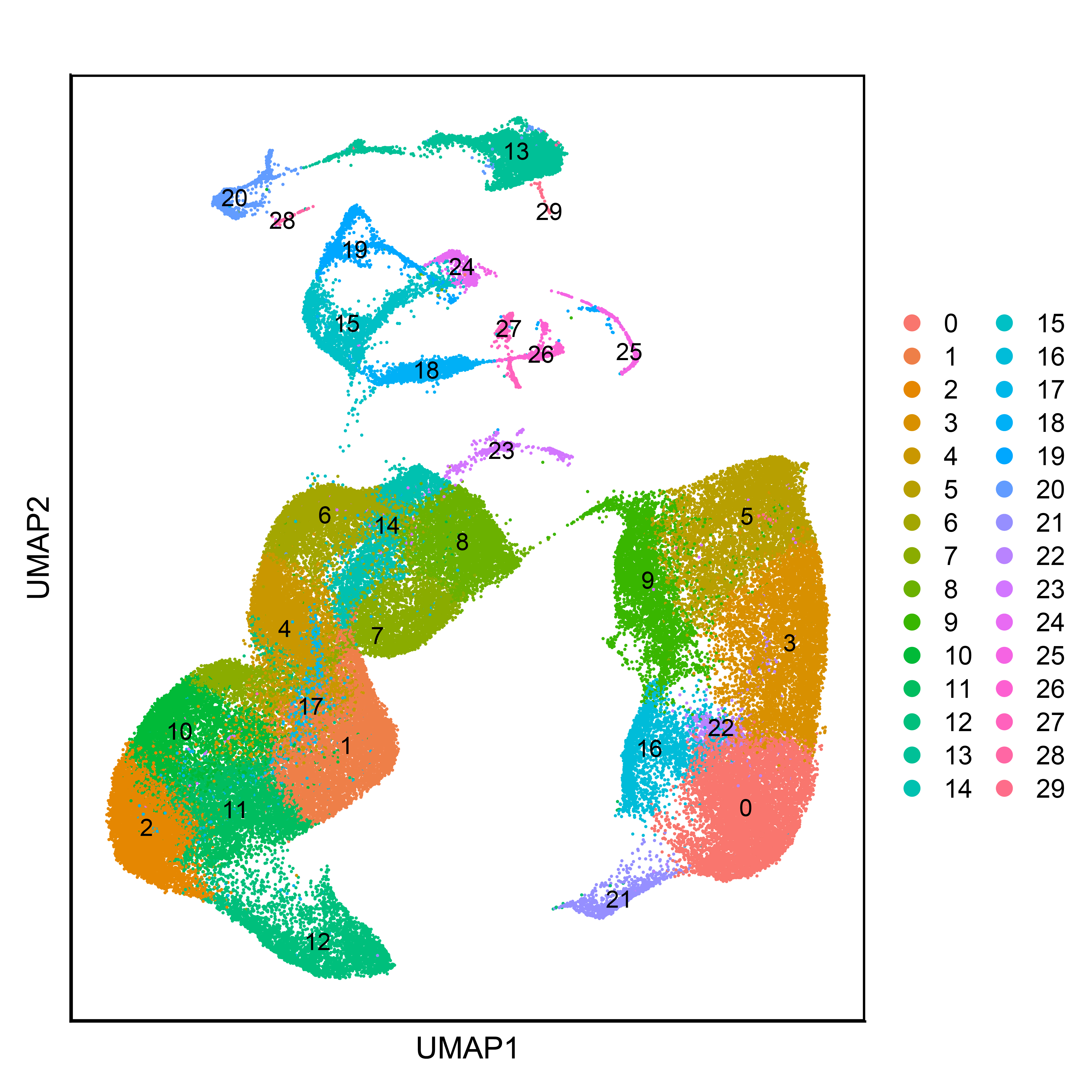

Supplement: Supplementary file 5 [file Image2.jpeg]

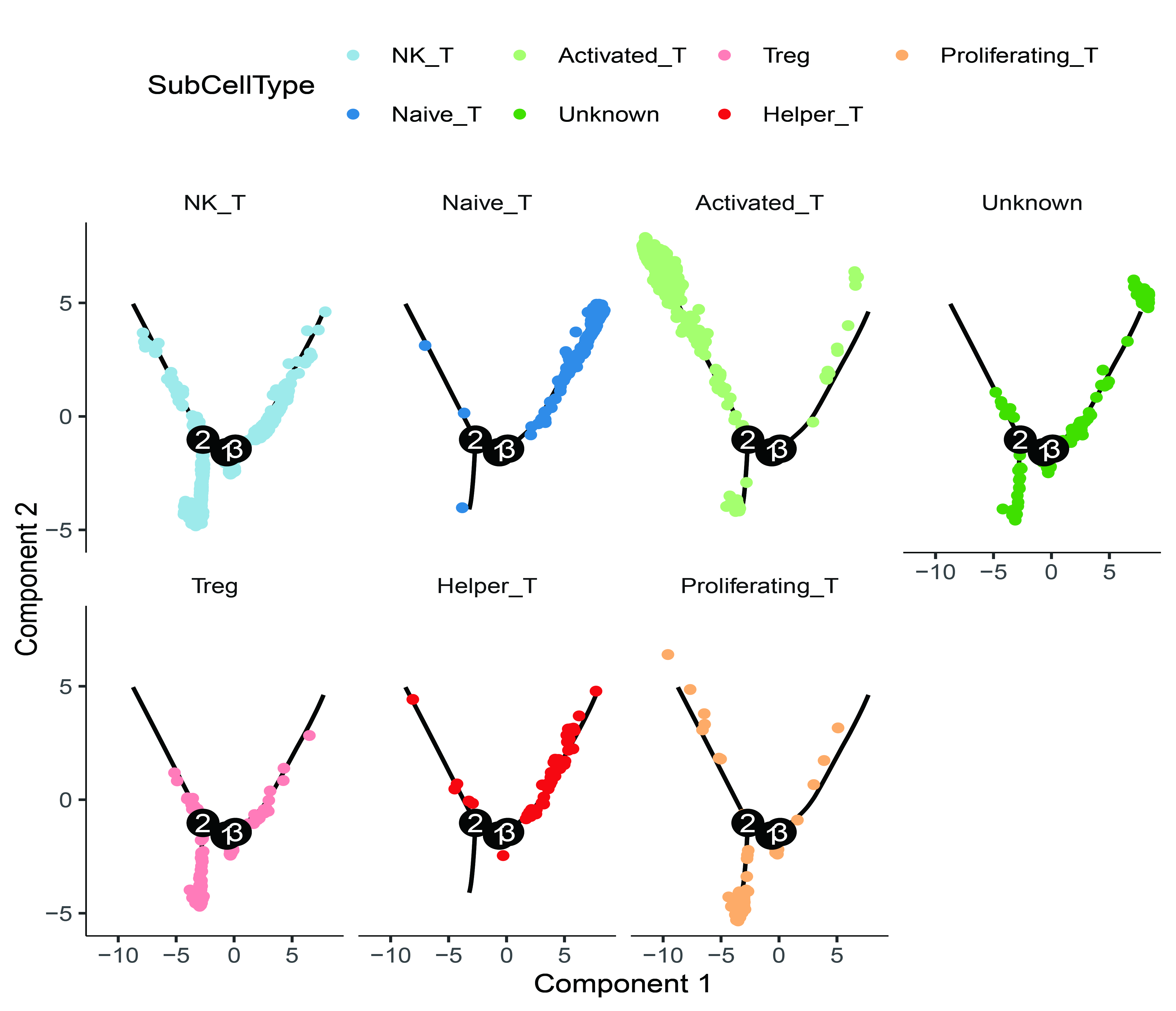

Supplement: Supplementary file 6 [file Image5.jpeg]
